# ^1^H NMR study of the interaction of *trans-*resveratrol with soybean phosphatidylcholine liposomes

**DOI:** 10.1038/s41598-019-54199-7

**Published:** 2019-11-28

**Authors:** Maria Cristina Cardia, Carla Caddeo, Francesco Lai, Anna Maria Fadda, Chiara Sinico, Michel Luhmer

**Affiliations:** 1 Dipartimento di Scienze della Vita e dell’Ambiente, Sezione di Scienze del Farmaco, University of Cagliari, CNBS, Via Ospedale 72, 09124 Cagliari, Italy; 2 Laboratoire de Résonance Magnétique Nucléaire Haute Résolution, Service de Chimie et PhysicoChimie Organiques, Université libre de Bruxelles (ULB), Avenue F. D. Roosevelt 50, CP160/08, 1050 Brussels, Belgium

**Keywords:** Self-assembly, Food nanotechnology

## Abstract

Resveratrol (RSV) is a well-known natural derivative with a wide range of biological and pharmacological activities. Despite of these demonstrated properties, it exhibits low both aqueous solubility and chemical stability and therefore low bioavailability. Consequently, the major concern of the technological research is to exploit delivery systems able to overcome bioavailability problems. In the recent past liposomes have been successfully studied for these purposes. In this paper, ^1^H-NMR spectroscopy, Nuclear Overhauser Spectroscopy (NOESY) as well as Paramagnetic Relaxation Enhancements (PRE) experiments have been carried out to quantitatively investigate the incorporation of resveratrol, at both the liposome preparation stage and by preformed liposomes, also with the aim to characterize resveratrol- soybean phosphatidylcholine (P90G) lipid bilayer interactions. Overall results of ^1^H NMR spectroscopy analysis suggest that RSV is located nearby the phosphocholine headgroups and also provide quantitative data on the incorporation of RSV (5% w/w), which corresponds to a 150-fold increase with respect to the solubility of RSV in water. Beside, considering that the same level of RSV incorporation was obtained via spontaneous uptake by preformed P90G liposomes, it can be concluded that RSV easily diffuses through the lipid bilayer.

## Introduction

**Figure 1:**
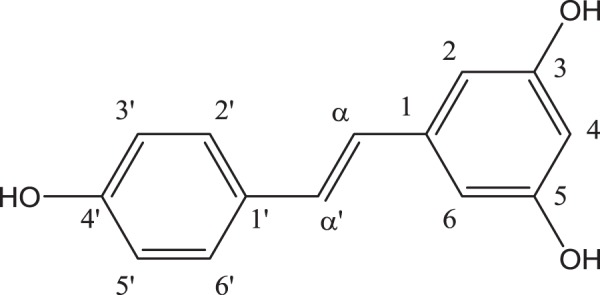
Structure and numbering of *trans*-resveratrol (RSV).

The plant-derived polyphenol *trans*-3,4′,5-trihydroxystilbene (Fig. 1), commonly named *trans*-resveratrol and simply referred to as RSV herein, has been the subject of intense and continuing research during the last decades, especially regarding its health benefits. About 5000 reports explicitly mentioning resveratrol in the title are found in PubMed for the period 2008–2017. About 600 of such reports were published during the first nine months of 2018, among which 41 reviews. RSV is a potent anti-oxidative agent and was reported to show a wide range of biological and pharmacological activities, including multi-targeted anti-microbial properties, anti-inflammatory, anti-cancer, anti-aging activities as well as neuro-protective and cardio-protective effects^1–3^. More than 70 plant species are known to produce RSV in response to environmental stress such as UV radiation, drought, parasitic or fungal attack. RSV can be found in common edible fruits, notably in grapes, blueberries, blackberries and peanuts. However, RSV exhibits low aqueous solubility and, consequently, poor absorption when administered orally. Furthermore, RSV also suffers from poor chemical stability. The search for effective delivery systems capable to overcome solubility, stability and bioavailability issues is therefore a major concern. Several approaches have been described, including the use of surfactants and co-solvents, particle size reduction down to nanocrystals (nanosizing), inclusion complex formation (notably with cyclodextrins), binding to transport proteins as well as loading in lipidic or polymeric nanosystems^4–8^. Among the nanocarriers, liposomes have demonstrated to incorporate RSV successfully, improving both biological activity and stability under UV exposure^9–11^.

Previous studies agree that RSV gives rise to non-specific interactions with phospholipid bilayers but the location of RSV remains unclear. Indeed, it has been either concluded that RSV is located in the hydrophobic chain region or, on the opposite, in the hydrophilic headgroup region. Bonechi *et al*. reported on liposomal RSV formulations made with a saturated phosphatidylcholine (DPPC = 1,2-dipalmitoyl-sn-glycero-3-phosphocholine) and either cholesterol (CHOL) or a positively charged derivative of cholesterol (DC-CHOL = 3ß-[N-(N’,N’-dimethylaminoethane)-carbamoyl]cholesterol)^12^. Several techniques were used for characterizing these formulations among which Electron Spin Resonance (ESR) and NMR. Interestingly, the authors pointed out marked differences in the location of the polyphenol compound, RSV being associated with the surface of the zwitterionic DPPC/CHOL liposomes but more deeply inserted in the bilayer of the cationic DPPC/DC-CHOL liposomes. Balanč *et al*. investigated the interaction of RSV with multilamellar vesicles prepared from purified soybean phosphatidylcholine (Phospholipon® P90NG). Based on ESR and fluorescence spectroscopy data, it was concluded that RSV is rather positioned in the inner part of the liposome membrane. These authors also highlighted the fact that RSV can penetrate from an external solution throughout an already formed lipid bilayer^13^. de Ghellinck *et al*. used neutron reflectometry to investigate the position of RSV in solid supported DPPC or DPPC/CHOL bilayers in their fluid state (at 335 K), immersed in an aqueous subphase. Their results notably reveal accumulation of RSV in between the phosphocholine headgroups, which consequently undergoes a conformational change, and exclude the presence of RSV in the hydrophobic core. For DPPC bilayers loaded by spontaneous insertion from the subphase, it was concluded that RSV accumulates in the outer headgroup region (facing the subphase) and that RSV is not transferred to the inner headgroup region (facing the solid support)^14^. Recently, Han *et al*. studied the influence of RSV on the membrane fluidity and polarity of three different types of unilamellar liposomes^11^. 1,2-dioleoyl-sn-glycero-3-phosphatidylcholine (DOPC) liposomes, DOPC/sphingomyelin/cholesterol liposomes and sphingomyelin/cholesterol liposomes of about 100 nm were prepared using the thin-film hydration followed by extrusion method. Using membrane-binding fluorescent probes they suggested a different RSV location in the liposome membranes. In the DOPC liposomes, characterized by less packed acyl carbon chains (liquid-disordered phase), RSV could penetrate in the lipid membrane and interacts with the membrane headgroup region more strongly than with the deeper parts of the bilayer. This RSV interaction and insertion into the bilayer determine an increase of the membrane stiffness. However, in the DOPC/sphingomyelin/cholesterol liposomes and sphingomyelin/cholesterol liposomes characterized by more packed acyl carbon chains (liquid-ordered phase and solid-ordered phase), RSV cannot penetrate into the lipid membranes and remains associated with the liposomes surface. Similar results were obtained by Neves *et al*. using unilamellar liposomes made of egg phosphatidylcholine, cholesterol, and sphingomyelin as model membranes^15–18^.

Pursuing our interest in RSV delivery systems and especially in liposomal RSV^9,19,20^, we recently reported preliminary ^1^H NMR results on the incorporation of RSV by soybean phosphatidylcholine liposomes. This study convinced us that ^1^H NMR, which is not so frequently used for the characterization of drug-liposomes interactions, could provide detailed information on the incorporation of RSV by such liposomes and notably on the location of RSV^19^. Herein, we report the results of various ^1^H NMR experiments, among which are titration experiments, NOESY (Nuclear Overhauser Effect SpectroscopY) experiments as well as PRE (Paramagnetic Relaxation Enhancement) experiments, aimed at investigating quantitatively the incorporation of RSV, at both the liposome preparation stage and by preformed liposomes, and also with the purpose to characterize the interactions of RSV with the lipid bilayer.

## Materials and Methods

### Materials

*Trans*-resveratrol (RSV, >99% pure, M.W. 228.25) was purchased from Galeno (Italy); methanol-*d*_4_, D_2_O (99.9 atom % D) and potassium hexacyanochromate(III) were purchased from Sigma-Aldrich.

Phospholipon® 90 G (P90G, M. W. of approximately 776), *i.e*. soybean phosphatidylcholine with a purity of minimum 94%, was supplied by Lipoid (Germany). The ^1^H NMR spectrum of P90G dissolved in methanol-*d*_4_ is described in the Supplementary Material (Figure S1). The integrals agree with the expected acyl chain distribution and the corresponding average molecular weight is 775 g/mole.

### Liposome preparation and size and morphology characterization

P90G (100 mg/ml) liposomes were prepared in pure D_2_O, typically in 2 mL batches, by a sonication method previously established^9^. Briefly, the components were weighed in a glass flask and D_2_O was added. The dispersion was sonicated using a high intensity ultrasonic disintegrator (Soniprep 150, MSE Crowley, London, UK), with the probe directly immersed in the sample. The sonication protocol consisted in the following two consecutive series of on–off cycles using a probe amplitude of 13 µm: (i) 20 cycles with 5 s on and 2 s off, (ii) 30 cycles with 3 s on and 2 s off. The samples were kept in the fridge at about 277 K until the NMR analyses, which typically took place the day after preparation. Dynamic Light Scattering (DLS) analyses were carried out at room temperature, after 1:100 dilution in H_2_O, using a Zetasizer Nano-ZS apparatus (Malvern Instruments, Worcestershire, UK).

The liposomes structure was visualized by using cryo-transmission electron microscopy (cryo-TEM). The sample was diluted with water and applied onto lacey carbon film grids (300 microMesh, EMS). The grid was blotted in an automatic plunge freezing apparatus (Thermo Fisher, Hillsboro, USA) to control humidity and temperature. Observation was made at 103 K in a Tecnai F 20 microscope (Thermo Fisher, Hillsboro, USA) operating at 200 kV, equipped with a cryo-specimen holder Gatan 626 (Warrendale, PA, USA). Images were collected at different magnifications using a defocus range from −1 to −2.5 µm with an electron dose of 30 e^−^/Å^2^. Digital images were drift corrected using a Falcon III camera (Thermo Fisher, Hillsboro, USA) 4096 × 4096 pixels.

### NMR measurements

The NMR spectra were recorded on a Varian Unity 500 spectrometer equipped with an indirect detection dual probe and temperature regulation. The 1D ^1^H spectra were recorded at 300 K using the following acquisition parameters: 10 s relaxation delay, 6.4 µs high-power RF pulse-width (flip angle of 90°), 2 s acquisition time, 24 ppm spectral width centered at about 5 ppm, 32 repetitions. The processing comprised zero-filling (total of 32k points) and exponential multiplication of the free induction decay (line broadening factor lb = 2 Hz for liposome samples), Fourier transform, phase correction and chemical shift referencing of the spectrum. The signal of the solvent (HDO) was used as internal standard and set to 4.746 ppm for spectra recorded at 300 K^21^. Molar concentration data determined by ^1^H NMR were obtained from integrated signal intensities measured for the sample of interest and for an external standard of known concentration in D_2_O. The spectrum of this standard was recorded the same day, using exactly the same acquisition parameters as for the spectrum of the studied sample. The same processing was applied to both spectra and the integrals were determined by deconvolution (best-fit analysis). Details on the titration experiments and on the deconvolution analysis are given in the Supplementary Material.

### Statistical analysis of data

Instat software (GraphPad Prism4, San Diego, CA) was used to analyze the data. One-way analysis of variance (ANOVA) with post hoc analysis using Tukey’s multiple comparison test was used for parametric data. The results of multiple observations were presented as the means ± S.D of at least 3 separate experiments. A p value lower than 0.05 was considered statistically significant.

## Results and Discussion

### DLS and cryo-TEM analyses

**Figure 2:**
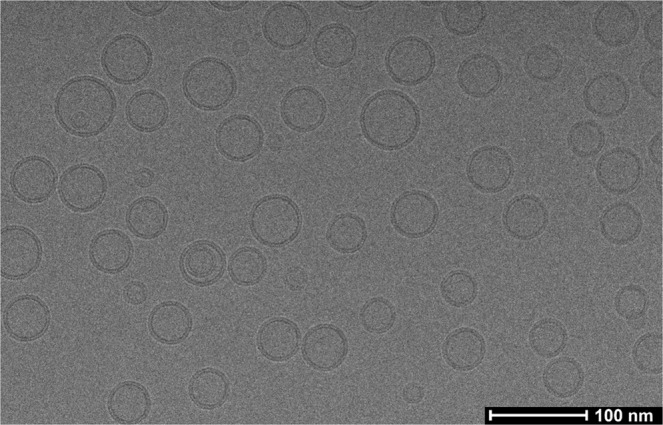
Cryo-TEM micrograph of 2 mg/ml RSV loaded P90G liposomes.

Samples containing 100 mg of soybean phosphatidylcholine (P90G) and various amounts of RSV (0, 2, 4, 6 or 8 mg) for 1 mL of D_2_O were prepared as described in the section Material and Methods. As reported in literature^19^, and demonstrated by cryo-TEM images, the preparation method used in this work allowed us to obtain small unilamellar vesicles (SUV) (Fig. 2). The samples of plain liposomes and those prepared with 2 or 4% of RSV were visually homogenous, fluid and slightly opalescent. In contrast, gel-like and opaque white samples were obtained with 6 or 8% of RSV. The DLS average size, polydispersity index and zeta potential of the different liposome formulations are reported in Table 1. It must be stressed that the samples were first diluted with water before DLS analysis (1:100 dilution) and were all visually homogenous. The DLS data in Table 1 highlight that the average size of the liposomes prepared in this work increases with the RSV concentration and, on the other hand, that the polydispersity index decreases. Indeed, the presence of RSV not only can modify the bilayer fluidity and polarity but also other bilayer characteristics like the vesicle overall curvature: probably the presence of RSV modifies phospholipids aggregation, favoring the formation of higher diameter vesicles^12^.

**Table 1:** DLS characterization of the liposomal preparations.

RSV/P90G mass ratio (mg/mg)	RSV/P90G molar ratio (µmol/µmol)	Diameter (nm)	Polydispersity Index	Zeta potential (mV)
Plain liposomes^(a)^	Plain liposomes	70 ± 4	0.27 ± 0.01	−22 ± 2
2/100^(b)^	9/130	78 ± 2	0.26 ± 0.01	−25 ± 3
4/100^(c)^	18/130	91 ± 10	0.25 ± 0.01	−29 ± 1
6/100^(c)^	26/130	142 ± 7	0.17 ± 0.01	−34 ± 2
8/100^(c)^	35/130	173 ± 6	0.17 ± 0.01	−37 ± 1

Statistics on n independent samples: ^(a)^n = 5, ^(b)^n = 3, ^(c)^n = 2.

### RSV loading in P90G liposomes

**Figure 3:**
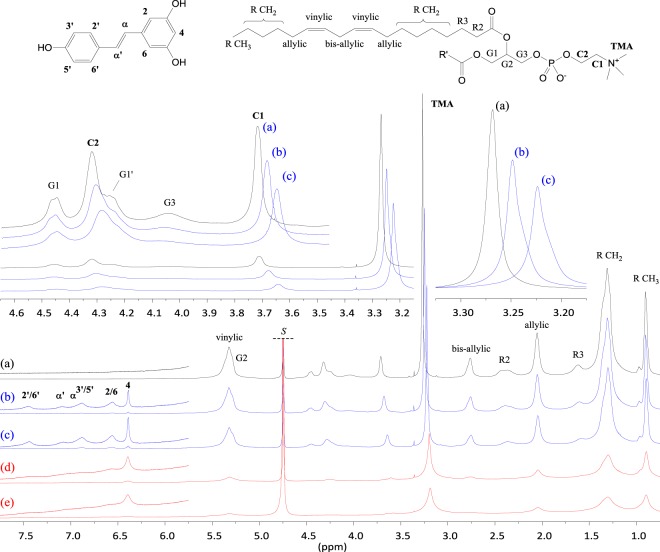
^1^H NMR spectra of 100 mg/mL P90G liposomes prepared in D_2_O with increasing amounts of RSV (300 K, 500 MHz). (**a**) in black: plain liposomes; (**b**) and (**c**) in blue: 2 and 4 mg/mL RSV respectively; (**d**) and (**e**) in red: 6 and 8 mg/mL RSV respectively. The signal of the solvent (*S*) is truncated.

Quantitative ^1^H NMR spectra were recorded at 300 K, *i.e*. well above the gel-to-liquid phase transition temperature of soybean phosphatidylcholine reported to be between 243 and 253 K^22^. Besides, the sonication protocol used in this work for the preparation of liposomes in D_2_O yields small unilamellar vesicles. Consequently, the ^1^H NMR signals of plain P90G liposomes are rather well resolved and most of them exhibit modest broadening (Fig. 3a and Figure S1 in the Supplementary Material). At first sight, the ^1^H NMR spectra of the samples prepared with 0, 2 or 4% w/w RSV are very similar (Fig. 3a–c). However, a closer look reveals the following changes in the presence of RSV: (i) the P90G signals are all slightly broadened (several Hz for the C1 signal, for instance), (ii) the trimethylammonium (TMA), C1 and C2 signals of the phosphocholine headgroup are weakly, but significantly, shifted toward lower frequencies (high-field shifted) and (iii) the TMA signal varies in shape and exhibits a shoulder. Very weak alterations were also detected for the G1 and G3 signals of the glycerol unit as well as for the R2 signal but not for the other signals of the acyl chains. In contrast, the ^1^H NMR spectra of the samples prepared with 6 or 8% of RSV exhibit important signal broadening (Fig. 3d,e). This could be due to increased stiffness of the bilayer but, considering the DLS results and the visual aspect of these samples, it is likely that it is primarily due to increasing particle-size and/or possibly aggregation. The ^1^H NMR signature of RSV agrees with the one reported for RSV interacting with DPPC-based liposomes (Fig. 3b)^12^. The signals of RSV are significantly broadened, with those of H-α and H-3′,5′ being superimposed at about 6.9 ppm. The signal of H-4 appears symmetric and is comparatively narrow, but it must be stressed that its full width at half maximum (FWHM) is about 10 Hz in Fig. 3b,c, 25 Hz in Fig. 3d and reaches 35 Hz in Fig. 3e. No significant chemical shift variation was observed for the signals of RSV.

**Figure 4:**
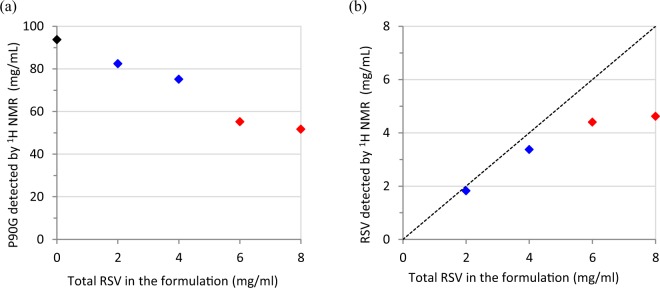
Amount of (**a**) phosphatidylcholine and (**b**) RSV detected by ^1^H NMR for samples of 100 mg/mL P90G liposomes prepared in D_2_O with increasing amounts of RSV (black: plain liposomes; blue: 2 and 4 mg/mL RSV; red: 6 and 8 mg/mL RSV, as in the corresponding spectra shown in Fig. 3). The dotted line in (**b**) is a guide for the eye and corresponds to the expected relationship if the entire amount of RSV was incorporated and detected.

The regions of the TMA and H-4 signals were submitted to a deconvolution analysis and the integrals were used to determine the amounts of P90G and RSV that are detected by ^1^H NMR, respectively (Fig. 4 and Supplementary Material). In the absence of RSV, more than 90% of the expected amount of phosphatidylcholine is detected. This figure decreases to about 75% for the sample prepared with 4 mg of RSV, which exhibits slightly larger average particle size (Table 1), and drops down to 55–50% for the samples prepared with 6 and 8 mg of RSV. It must be stressed that the integral measurement is more prone to systematic errors for the RSV-loaded samples. Indeed, as mentioned above, the TMA signal exhibits a shoulder in the presence of RSV and, therefore, two components plus local baseline correction were needed in the deconvolution procedure. Obviously, proper integral measurement of the TMA signal is even more difficult for the samples with 6 and 8 mg of RSV due to important signal broadening but it remains that the intensity loss is real. Indeed, the spectra also showed a growing background signal for increasing amount of RSV (see Figures S12 and S13), which is ascribable to ineffective motional narrowing and consistent with the formation of larger vesicles and/or aggregates. As regards RSV, the detected and expected amounts are in rather good agreement up to 4% of RSV (Fig. 4b). However, the amount of RSV detected by ^1^H NMR then reaches a plateau. All in all, these results strongly suggests that the maximum RSV loading in P90G liposomes is on the order of 5% w/w. Dispersion of excess solid RSV and aggregation phenomena probably occur above this threshold, yielding heterogeneous samples.

### Spontaneous uptake of RSV by P90G liposomes

**Figure 5:**
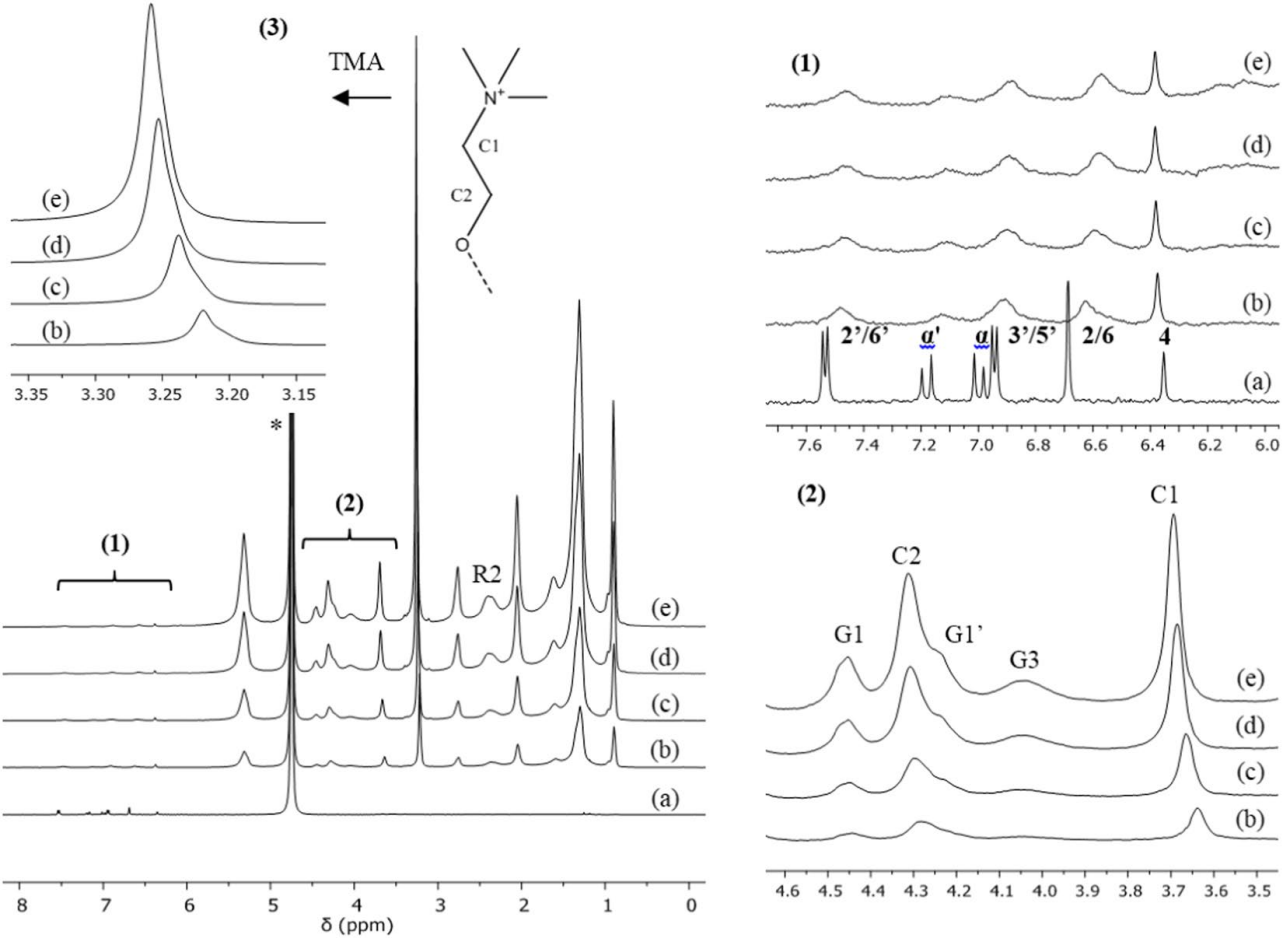
Titration of a saturated solution of RSV by plain P90G liposomes. ^1^H NMR spectra of (**a**) RSV in D_2_O and (**b**–**e**) mixtures of RSV with increasing amounts of P90G liposomes (300 K, 500 MHz). * The signal of the solvent is truncated. The sample used to acquire spectrum (**a**) comprised 600 µL of RSV solution. Spectra (**b**–**e**) were recorded after a total addition of 5, 10, 20 and 30 µL of a 100 mg/mL dispersion of plain P90G liposomes. (1) Region showing the signals of RSV (see Fig. 1 for assignment); the signals H-2,6 and H-4 are, respectively, unresolved doublet and triplet with a ^4^J scalar coupling of about 2.2 Hz. (2–3) Regions showing the signals C2, C1 and TMA (trimethylammonium) of the choline head group of P90G; signals G1/G1’ and G3 pertain to the methylene ^1^H of the glycerol unit.

A saturated solution of RSV (600 µL) was titrated by a 100 mg/mL dispersion of plain P90G liposomes (Fig. 5 and Figure S4). As expected, the ^1^H NMR spectra of RSV dissolved in D_2_O shows 6 narrow signals in the low field region, above 6 ppm, where no resonance of the liposomes is observed (Figure 5.1a). Analysis of the integrated intensities revealed a RSV concentration of 1.4 × 10^−4^ mol/L (300 K), in agreement with literature data for the solubility of RSV in water which typically range between 20 and 30 µg/mL at 298 K, *i.e*. between 0.9 and 1.3 × 10^−4^ mol/L^ 4,23–25^. Addition of 5 µL of the liposomal dispersion strongly affects the ^1^H NMR signature of RSV. Indeed, the RSV signals are strongly broadened, with the exception of the signal pertaining to H-4. In addition, significant upfield shifts are observed for all of them but, again, signal H-4 which is slightly downfield shifted. The integrated intensities are somewhat increased with respect to the spectrum recorded in the absence of P90G and this most probably originates from the presence of residual solid RSV in the initial sample. Further addition of liposomes has a much smaller impact on the signals of RSV and does not significantly affect their intensity (the corresponding RSV concentration is about 2.0 × 10^−4^ mol/L). As expected, the intensity of the P90G signals increases in the course of the titration. Again, significant chemical shift variations were observed for the TMA, C1 and C2 signals of the phosphocholine headgroup (Figures 5.2 and 5.3), which are shifted downfield toward the value measured in the absence of RSV. The TMA signal also varies in shape (Figure 5.3); it exhibits a shoulder which fades as the amount of liposomes increases. A control experiment confirmed that these observations are a consequence interactions between RSV and P90G liposomes. Indeed, no chemical shift variation was detected upon repeated additions of the liposomal dispersion into pure D_2_O and a symmetric TMA signal was observed (see Figures S5 and S6).

**Figure 6:**
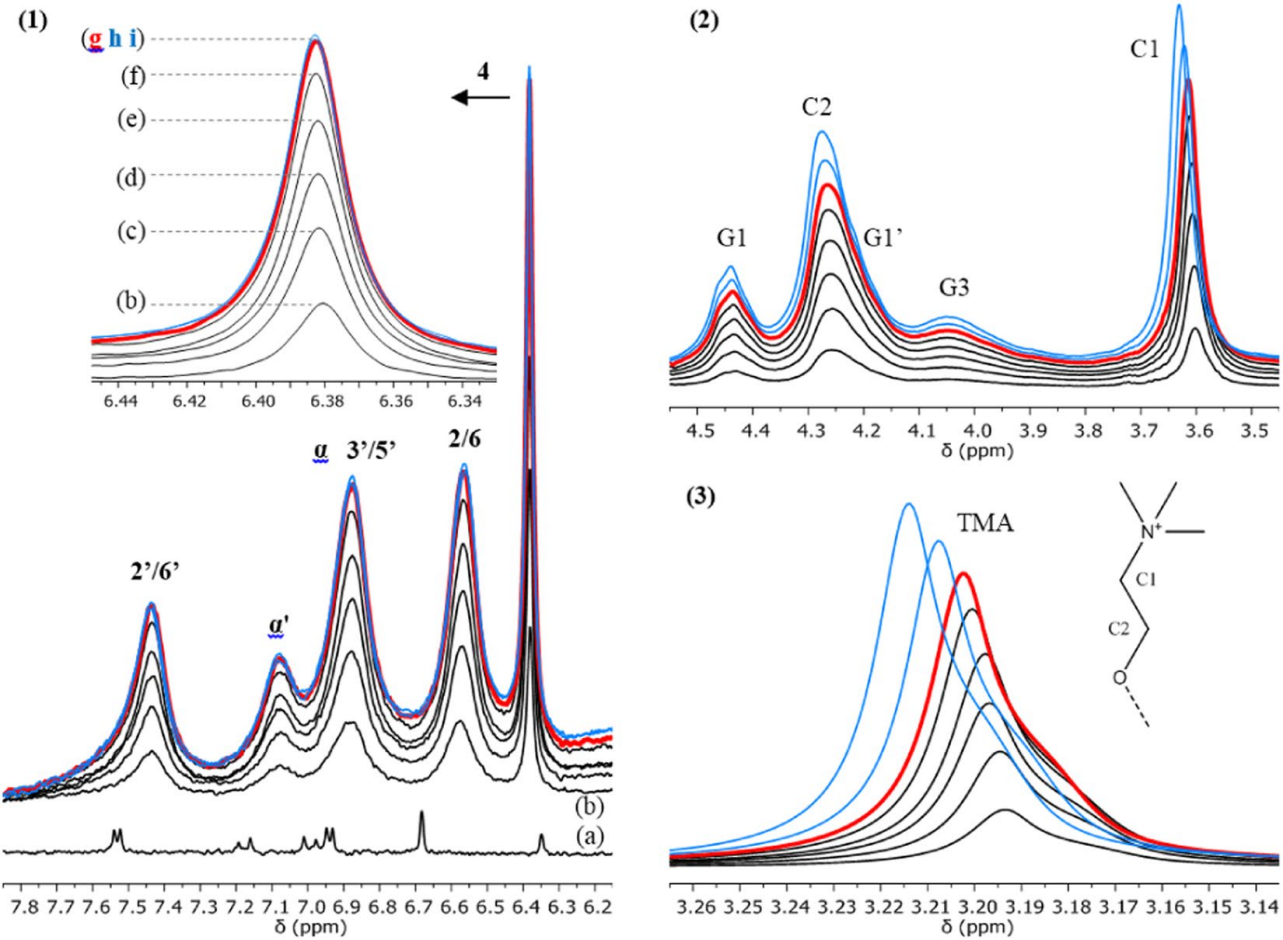
Titration of an excess of solid RSV in D_2_O by plain P90G liposomes. Regions of the ^1^H NMR spectra recorded (**a**) in the absence of P90G liposomes and (**b**–**i**), at the same scale, after mixing with increasing amounts of P90G liposomes (300 K, 500 MHz). (1) Region showing the signals of RSV. (2–3) Regions showing the signals C2, C1 and TMA of the choline head group of P90G. The sample used to acquire spectrum (**a**) comprised 500 µL of D_2_O and about 1 mg of RSV, which was mainly undissolved. Spectra (**b**–**i**) were recorded after repeated additions of 40 µL aliquots of a 100 mg/mL dispersion of plain P90G liposomes, up to a total addition of 320 µL; these spectra are superimposed without vertical offset. Spectrum (**g**) in red was recorded after a total addition of 240 µL; the two subsequent spectra (h – 280 µL and i – 320 µL) are in blue.

**Figure 7:**
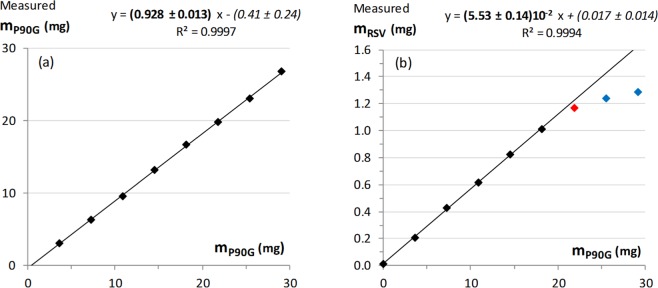
Titration of an excess of solid RSV in D_2_O by plain P90G liposomes. Variation of the amount of (**a**) phosphatidylcholine and (**b**) RSV detected by ^1^H NMR as a function of the total amount of P90G liposomes added in the sample. The red data point corresponds to a total addition of 240 µL and the blue data points correspond to the two subsequent additions of liposome dispersion (see Fig. 6). The confidence intervals correspond to twice the fitting errors.

The spontaneous uptake of RSV by P90G liposomes was assessed quantitatively using an heterogeneous NMR sample comprising a significant excess of solid RSV (between 1.0 and 1.5 mg) and 500 µL of D_2_O that was titrated by a 100 mg/mL dispersion of plain P90G liposomes (Fig. 6 and Supplementary Material). As described above, the ^1^H NMR signature of RSV is strongly altered by the first addition of liposomes (spectra a and b of Figure 6.1). The intensity of the RSV signals increases significantly and goes on increasing upon further additions of liposomal dispersion up to a total addition of 240 µL (spectrum g of Figure 6.1, in red). In contrast, the two subsequent additions of liposomes do not significantly affect the intensity of the ^1^H NMR signals of RSV (spectra h and i, in blue). No significant chemical shift or linewidth variation was observed for the signals of RSV in the presence of increasing amount of liposomes (spectra b to i). As expected, the intensity of the P90G signals increases after each addition (Figures 6.2 and 6.3). The TMA signal exhibits two overlapping components in the whole range of P90G concentration. No significant linewidth variation is detected, suggesting that no important aggregation of liposomes occurs in the presence of excess solid RSV (see also Figure S11). At the beginning of the titration, negligible or weak chemical shift variations are observed for the C1, C2 and TMA signals; they are however more pronounced at the end of the experiment. The regions of the H-4 and TMA signals were submitted to a deconvolution analysis and the integrals were used to determine the molar concentration and the mass of the corresponding species detected by ^1^H NMR (Figure S10). The amount of phosphatidylcholine measured in this way varies linearly with the total amount of P90G liposomes added in the sample (Fig. 7a). The slope was found to be 0.93 ± 0.01, which is a very good result considering the possible sources of systematic errors. It validates the quantitative analysis of the NMR spectra and, most importantly, shows that the observed integral of the TMA signal is indicative of the actual amount of P90G liposomes in the sample and this for the entire titration experiment. Besides, it corroborates the absence of aggregation after mixing with excess solid RSV.

The amount of RSV detected by ^1^H NMR increases linearly at the beginning of the titration and reaches a maximum of about 1.3 mg (Fig. 7b), in agreement with the total amount of solid RSV introduced in the NMR tube. These results clearly indicate that the spontaneous uptake of RSV by P90G liposomes is an effective process. From the slope of the linear variation observed for the molar concentration data, it can be concluded the RSV/P90G ratio corresponding to saturation is about 20 mol%. Similarly, the slope of the linear variation observed for the mass data indicates that the maximum uptake of RSV is about 5.5% w/w (Fig. 7b).

### Location of RSV in P90G liposomes

The chemical shift and lineshape variations discussed in the two previous sections, which were observed for the TMA, C1 and C2 signals as well as for the G1, G3 and R2 signals but to a lesser extent, strongly suggest that RSV interacts with the phosphocholine headgroups of the bilayer rather than with the buried acyl chains.

**Figure 8:**
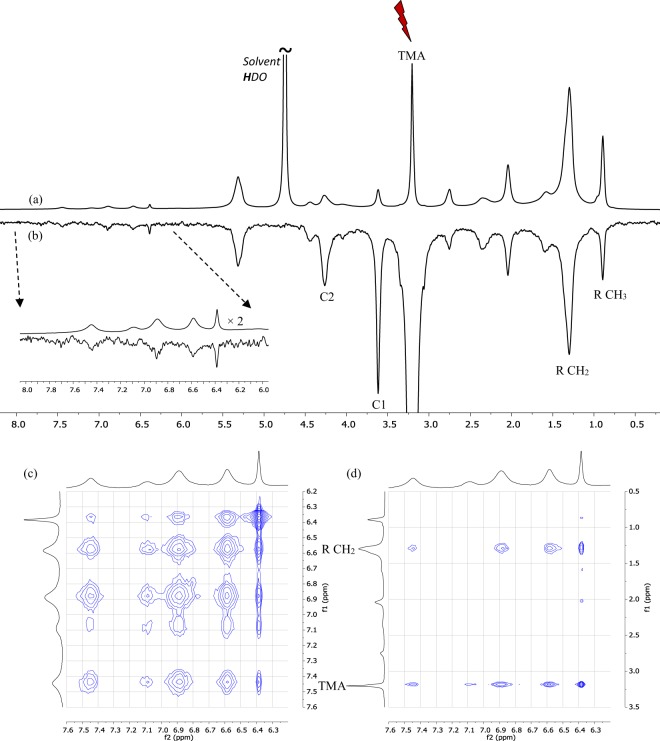
NOESY experiments (300 K, 500 MHz). NMR spectra recorded for a sample obtained by further addition of about 1 mg of solid RSV and 15 µL of a 100 mg/mL stock dispersion of plain P90G liposomes in the last sample used in the titration of the saturated solution of RSV (i.e. in the sample corresponding to spectrum (**e**) of Fig. 5). (**a**) equilibrium ^1^H spectrum, (**b**) NOESY-1D spectrum recorded with selective excitation at the frequency of the TMA signal of P90G and a mixing time of 200 ms, (**c**) and (**d**) regions of a NOESY-2D spectrum recorded with a mixing time of 200 ms.

Nuclear Overhauser Effects (NOE’s), which are a consequence of short-ranged dipolar interactions between nuclear spins, are commonly used for probing spatial proximity between ^1^H nuclei and it was reported that 2D-NOESY experiments provide information on the location of RSV in DPPC-based liposomes^12^. Cross-peaks were observed between all the ^1^H signals of RSV and both the ^1^H signal of the CH_2_ groups of the acyl chains (R CH_2_ signal) and the signal of the CH_3_ groups of the DPPC polar-head (TMA signal). Based on this, the authors concluded that RSV is deeply inserted in the bilayer. In the present work, both 1D and 2D NOESY experiments were conducted (see Supplementary Material). A NOESY-1D spectrum was recorded with selective excitation at the frequency of the TMA signal and revealed negative enhancements for the signals of RSV, which indicates slow tumbling and is thus consistent with RSV interacting with the liposomes (Fig. 8a,b). All the signals of RSV were affected to a similar extent, indicating that no specific NOE was observed. Furthermore, in addition to the effects expected for the proximal C1 and C2 ^1^H of the phosphocholine headgroup, the spectrum also revealed negative enhancements for all the signals of P90G, including the signal of the remote methyl groups of the acyl chains (R CH_3_ signal). This is a consequence of very slow tumbling motion and thus effective spin-diffusion, which prevents the use of NOE’s for probing spatial proximity. This was confirmed by a NOESY-2D experiment, which yielded similar results as those mentioned above for DPPC-based liposomes but also revealed negative cross-peaks between H-4 and all the other signals of RSV (Fig. 8c,d). Hence, no information regarding the location of RSV in the bilayer can be gained from NOESY experiments.

**Figure 9:**
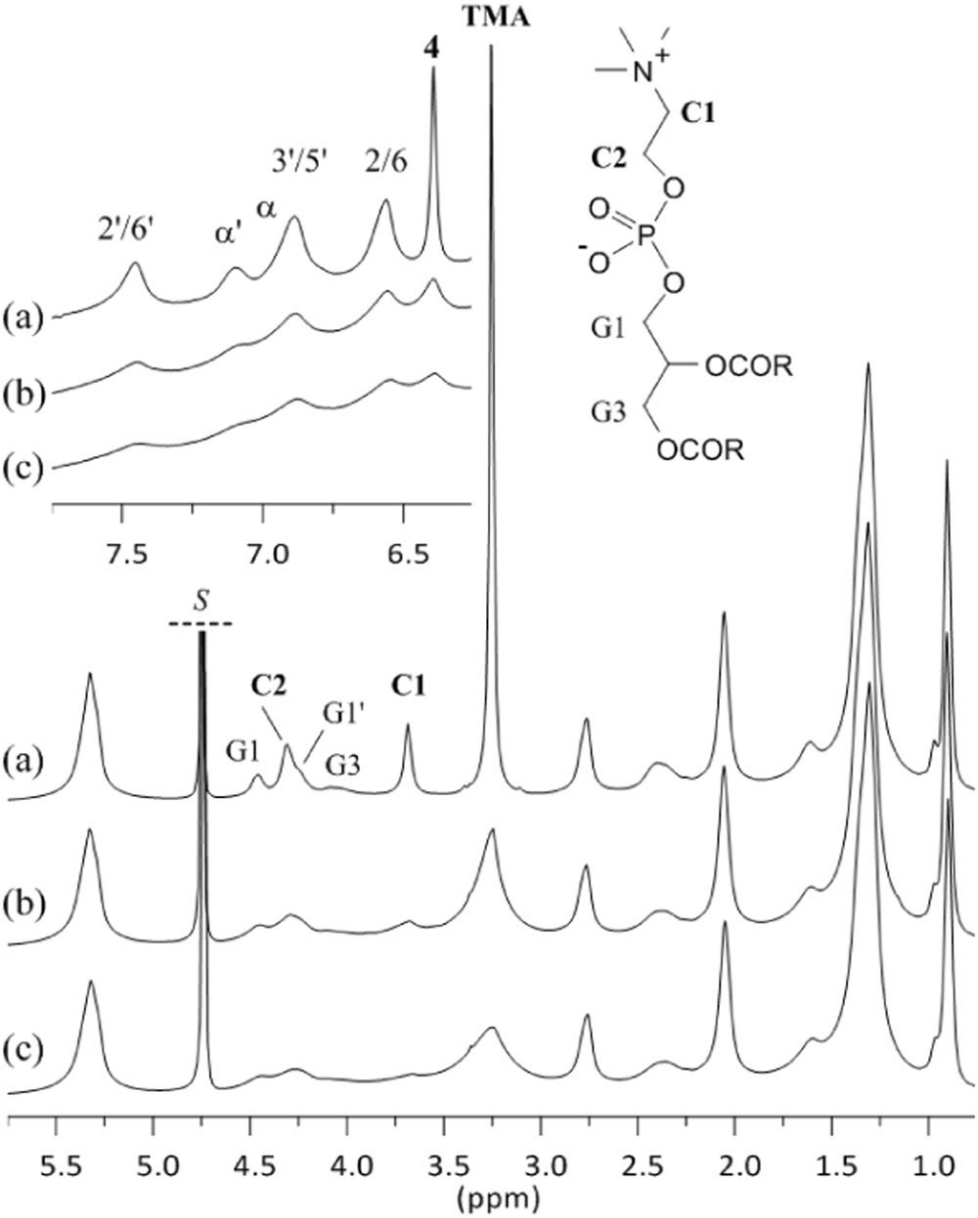
^1^H NMR spectra of RSV-loaded P90G liposomes (100 mg/mL lipid, 2% w/w RSV) prepared in D_2_O with increasing amounts of potassium hexacyanochromate(III): (**a**) no paramagnetic salt, (**b**) 1.9 mM and (**c**) 4.5 mM K_3_[Cr(CN)_6_].

Paramagnetic relaxation enhancements (PRE’s), which originates from electron-nucleus dipolar interactions, are a valuable alternative to obtain structural information by NMR, especially for large biomolecular systems^26^. The experiments reported in the present work rely on the use of the hexacyanochromate(III) anion as paramagnetic probe, which has been successfully exploited to characterize micellar incorporation, and focus on transverse relaxation enhancements that are easily detected in the 1D ^1^H NMR spectrum as additional signal broadening^27^. Samples of RSV-loaded liposomes (100 mg/mL P90G, 2% w/w RSV) containing K_3_[Cr(CN)_6_] were prepared in D_2_O following the same procedure as before (see Materials and Methods); the paramagnetic probe was thus present in both the outer and inner aqueous media of the liposomes. As shown in Fig. 9, the ^1^H NMR signals of the phosphocholine polar head (TMA, C1 and C2 signals) exhibit significant broadening in the presence of the paramagnetic salt whereas no significant transverse PRE are detected for the ^1^H of the acyl chains. This is consistent with the fact that the paramagnetic anion [Cr(CN)_6_]^3−^ cannot penetrate the lipid bilayer and therefore induces distance-dependent PRE effects relative to the liposome outer and inner surfaces. The spectra also reveal significant broadening of the signals of RSV, notably for the H-4 signal, indicating that RSV is not buried into the bilayer.

## Conclusion

**Figure 10:**
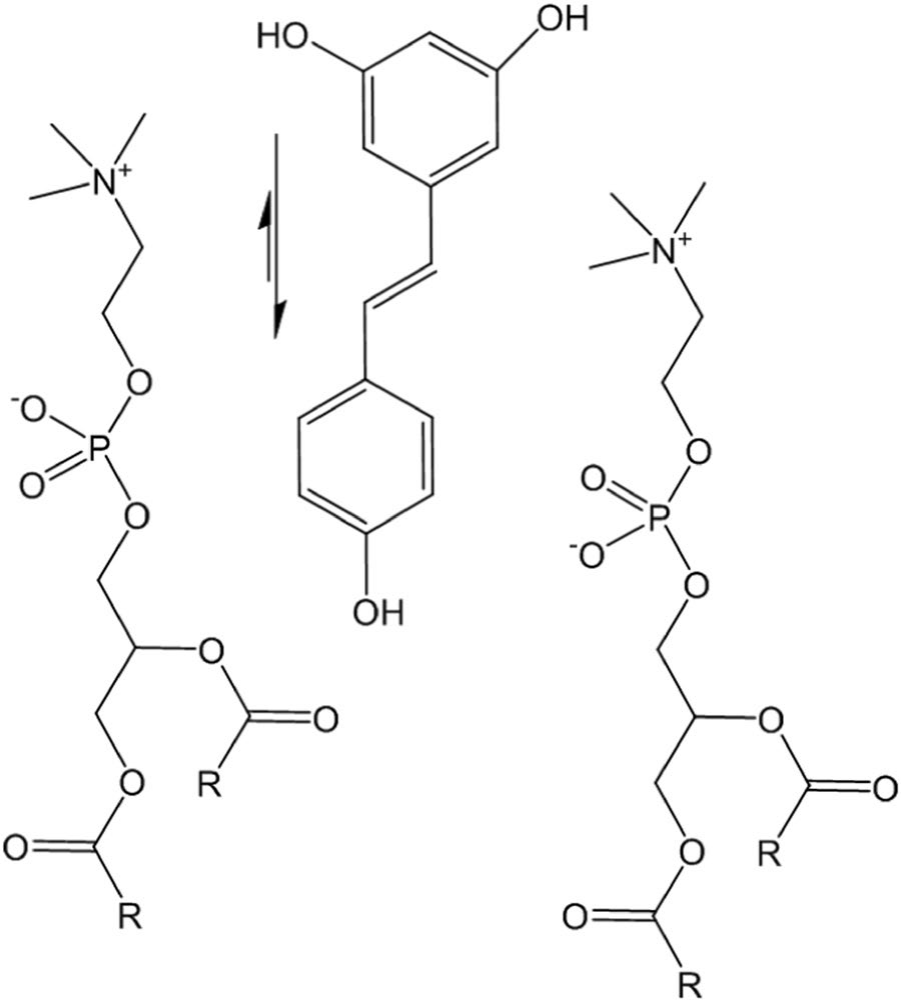
Representation of the preferential location and expected orientation of RSV interacting with an aqueous dispersion of P90G liposomes.

Chemical shift and lineshape variations observed for the ^1^H NMR signals of P90G as well as transverse PRE data suggest that RSV is located nearby the phosphocholine headgroups as stated by Bonechi, de Ghellinck, Han and Neves but for different membrane models (lipids, cholesterol, vesicles structure) and using different investigation techniques^11,12,14,15,18^. Furthermore, our results show that the interaction of RSV with P90G liposomes has little effect on the chemical shift of H-4 while significant broadening due to transverse PRE was observed for this signal. It suggests that H-4 remains more exposed to the solvent, which in turn suggests that RSV adopts the preferential orientation depicted in Fig. 10. Even though speculative, this is consistent with the higher hydrophilicity of the 1,3-dihydroxybenzene segment, as indicated by the higher water solubility of resorcinol compared to phenol (about 720 and 85 g/L at 298 K, respectively)^28^.

The ^1^H NMR analyses reported in this work also provided quantitative data on the incorporation of RSV. The maximum loading achieved in the preparation of homogenous dispersions of small P90G liposomes was about 5% w/w at 300 K. Hence, for the 100 mg/mL liposomial formulations used in this work, the amount of RSV that can be incorporated corresponds to a molar concentration of about 20 mM, *i.e*. a 150-fold increase with respect to the solubility of RSV in water. Beside, considering that the same level of RSV incorporation was obtained via spontaneous uptake by preformed P90G liposomes, it can be concluded that RSV easily diffuses through the lipid bilayer, in agreement with the conclusion of Balanč^13^.

## Supplementary information

Supplementary Material
