# Supplementary material for: 1H NMR study of the interaction of trans-resveratrol with soybean phosphatidylcholine liposomes
Source: Sci Rep. 2019 Nov 28;9:17736. doi: 10.1038/s41598-019-54199-7 (PMC6883048; doi:10.1038/s41598-019-54199-7)
Supplement: Supplementary file 1 — Supplementary Material [file 41598_2019_54199_MOESM1_ESM.docx]

**Supplementary material**

**^1^H NMR study of the interaction of *trans-*resveratrol with soybean phosphatidylcholine liposomes**

Maria Cristina Cardia^a^, Carla Caddeo^a^, Francesco Lai^a^, Anna Maria Fadda^a^, Chiara Sinico^a^* and Michel Luhmer^b^*

*^a^ Dipartimento di Scienze della Vita e dell’Ambiente, Sezione di Scienze del Farmaco, University of Cagliari, CNBS, Via Ospedale 72, 09124 Cagliari, Italy.*

^b^ Laboratoire de Résonance Magnétique Nucléaire Haute Résolution, Service de Chimie et PhysicoChimie Organiques, Université libre de Bruxelles (ULB), Avenue F. D. Roosevelt 50, CP160/08, 1050 Brussels, Belgium

**Assignment of the ^1^H NMR signals of Phospholipon^®^ 90G**

About 5 mg of Phospholipon^®^ 90G (P90G, soybean phosphatidylcholine supplied by *Lipoid* with a purity of minimum 94%) were dissolved in 650 µL of methanol-*d_4_* and the solution was transferred in a standard 5 mm NMR tube. The NMR spectra were recorded at 298 K, without sample spinning, on a Varian VNMRS spectrometer operating at 14.1 T (600 MHz for ^1^H) and equipped with a standard 5 mm triple inverse z-gradient probe. The signal of the solvent was used for chemical shift referencing (^1^H signal of CHD_2_OD at 3.31 ppm and ^13^C signal of CD_3_OD at 49.00 ppm; *Gottlieb et al. J. Org. Chem. 1997, 62, 7512-7515*).

The ^1^H NMR spectrum of P90G in methanol-*d_4_* is shown in Figure S1, where it is compared to the corresponding spectrum of plain P90G liposomes directly prepared in pure D_2_O by sonication (see material and methods). The assignment was deduced from the analysis of the two-dimensional spectra shown in Figures S2-S3. Soybean phosphatidylcholine mainly comprises linoleic acid (18:2) in addition to several other fatty acids. The ^1^H NMR signal integrals calculated from the fatty acid distribution are in nice agreement with the experimental data (Table S1).

| Fatty acid distribution reported by *Avanti Polar Lipids*:  f_16:0_ = 14.9%, f_18:0_ = 3.7%, f_18:1_ = 11.4%, f_18:2_ = 63.0%, f_18:3_ = 5.7%, unknown 1.2% | | |
| --- | --- | --- |
| Signal | Calculated | ^1^H NMR spectrum |
| vinylic | 2×(2×f_18:1_ + 4×f_18:2_ + 6×f_18:3_)  **6.18** | **6.05** |
| allylic | 2×(4×f_18:1_ + 4×f_18:2_ + 4×f_18:3_)  **6.41** | **6.49** |
| bis-allylic 18:2 | 2×(2×f_18:2_)  **2.52** | **2.41** |
| bis-allylic 18:3 | 2×(4×f_18:3_)  **0.46** | **0.48** |
| R CH_3_ 18:3 | 2×(3×f_18:3_)  **0.34** | **0.35** |

**Table S1**: Fatty acid distribution *(*[*https://www.avantilipids.com*](https://www.avantilipids.com)*; catalog number 441601)* and ^1^H NMR signal integrals with respect to the integral of the trimethylammonium signal set to 9 (see Figure S1).

~

~

~

~

~

18:3

18:3

vinylic

R3

R2

allylic

bis-allylic

TMA

C2

C1

G3

G1’

G1

G2

R CH_2_

R CH_3_

*Solvent*

*C****H****D_2_OD*

*Solvent*

*CD_3_O****H***

TMA

*Solvent*

***H****DO*

**Figure S1**: ^1^H NMR spectrum of, above, P90G dissolved in CD_3_OD (298 K, 600 MHz, lb = 0.3 Hz) and, below, a 100 mg/mL dispersion of plain P90G liposomes in D_2_O (300 K, 500 MHz, lb = 2.0 Hz). The signal of the solvent was used for chemical shift referencing (δ_CHD2OD_ = 3.31 ppm at 298 K and δ_HDO_ = 4.746 ppm at 300 K; *Gottlieb et al. J. Org. Chem. 1997, 62, 7512-7515*).

**Figure S2**: Absolute-value dqfCOSY spectrum of P90G dissolved in CD_3_OD (298 K, 600 MHz).

*Solvent*

**Figure S3**: Regions of the edited HSQC (above) and 8Hz-HMBC (below) ^1^H-^13^C spectra of P90G dissolved in CD_3_OD (298 K, 600 MHz).

**Titration of a saturated solution of RSV by plain P90G liposomes and control experiment.**

About 4 mg of RSV and 2 mL of D_2_O were vortexed at room temperature for a few min; the sample was then centrifuged for 10 min at 14000 rpm and the supernatant was collected. 600 µL of this RSV solution, or 600 µL of pure D_2_O for the control experiment, were introduced into a standard 5 mm tube and submitted to the ^1^H NMR analysis. 5 or 10 µL aliquots of a 100 mg/mL stock dispersion of plain P90G liposomes were then precisely added in the NMR tube using a 20 µL - micropipette. After each addition, the sample was gently agitated, manually, for a few min and let at least 5 min into the NMR magnet for temperature stabilization prior to recording the spectrum. The ^1^H NMR spectra were recorded using a 90° pulse (ranging between 6.1 and 6.4 µs), an acquisition time of 2 s, a relaxation delay of 8 s, 32 scans in the absence of liposomes and 64 scans otherwise. Exponential apodization of the free induction decay with a line broadening factor (lb) of 2 Hz was applied before Fourier transform.

The series of spectra recorded for increasing amounts of P90G liposomes added in the RSV solution or in D_2_O (control experiment) are shown in Figures S4 and S5, respectively *(region showing the signals of the liposomes; see article for the region of the RSV signals)*.

The ^1^H NMR spectra recorded after addition of 5 µL of the stock liposome dispersion in the RSV solution or in D_2_O are compared in Figure S6.

~

TMA

*Solvent*

***H****DO*

(e)

(d)

(c)

(b)

(a)

R2

(e)

(d)

(a)

(b)

(c)

C1

C2

G1

G3

G1’

**Figure S4: Titration of a saturated solution of RSV by plain P90G liposomes.**

Region of the ^1^H NMR spectra, showing the signals of P90G, recorded for increasing amounts of plain P90G liposomes added in a saturated solution of RSV (300 K, 500 MHz). Spectra (a-e) were recorded after a total addition of 0, 5, 10, 20 and 30 µL of a 100 mg/mL stock liposome dispersion in 600 µL of the RSV solution; see Figure S1 for complete signal assignment.

~

*Solvent*

***H****DO*

(a)

(c)

(e)

(d)

(b)

TMA

R2

(a)

(b)

(c)

(d)

(e)

G3

G1

G1’

C2

C1

**Figure S5: Control experiment.**

^1^H NMR spectra recorded for increasing amounts of plain P90G liposomes added in D_2_O (300 K, 500 MHz). Spectra (a-e) were recorded after a total addition of 0, 5, 10, 20 and 30 µL of a 100 mg/mL stock liposome dispersion in 600 µL of D_2_O; see Figure S1 for complete signal assignment.

~

*Solvent*

***H****DO*

TMA

(b)

(a)

R2

G3

G1’

G1

C2

C1

**Figure S6: Titration of a saturated solution of RSV by plain P90G liposomes and control experiment.**

Region of the ^1^H NMR spectra, showing the signals of P90G, recorded after addition of 5 µL of a 100 mg/mL stock liposome dispersion, (a) below in black, in the saturated sample of RSV and, (b) above in blue, in D_2_O (300 K, 500 MHz) ; see Figure S1 for complete signal assignment.

**Uptake of RSV by P90G liposomes: qualitative results and NOESY experiments.**

About 1 mg of solid RSV and 15 µL of a 100 mg/mL stock dispersion of plain P90G liposomes were successively added to the last sample used in the titration of the saturated solution of RSV. This heterogeneous sample, comprising an excess of solid RSV and a total of 45 µl of stock P90G liposome dispersion, was thoroughly mixed using a vortex; this yields an opaque white suspension. The ^1^H NMR spectrum was recorded and processed as before. It is shown in Figure S7 together with the spectrum recorded before addition of solid RSV.

A NOESY-1D spectra was recorded with selective excitation at the frequency of the TMA signal of P90G, using a 180° seduce pulse shape (duration of 7.2 ms for a bandwidth of 120 Hz), a mixing time of 200 ms and 128 scans. It is shown in Figure 7 of the manuscript together with the equilibrium ^1^H spectrum (the spectra shown in this figure were processed with a line broadening factor lb = 5 Hz). A NOESY-2D spectrum was recorded using a mixing time of 200 ms; regions of this spectrum are also shown in Figure 7 of the manuscript.

TMA

~

*Solvent*

***H****DO*

(a)

(b)

**2’/6’**

**α'**

**α**

**3’/5’**

**2/6**

**4**

R2

(b)

(a)

G1’

C2

C1

G1

G3

**Figure S7: Uptake of RSV by P90G liposomes: qualitative results.**

^1^H NMR spectra recorded (a), in black, after addition of 30 µL of a 100 mg/mL stock dispersion of plain P90G liposomes in the saturated sample of RSV and (b), in red, for an heterogeneous simple obtained by further addition of about 1 mg of solid RSV and 15 µL of the stock liposome dispersion (300 K, 500 MHz).

**Titration of an excess of solid RSV in D_2_O by plain P90G liposomes.**

500 µL of D_2_O were added in a standard 5 mm NMR tube containing a small amount of solid RSV (between 1.0 and 1.5 mg); the sample was vortexed, decanted for about 2 hours at room temperature and then submitted to the ^1^H NMR analysis. 40 µL aliquots of a 100 mg/mL stock dispersion of plain P90G liposomes were precisely added into the NMR tube using a 100 µL - micropipette. After each addition, the sample was thoroughly agitated, first manually then using a vortex for at least 1 min. The first three additions yield an opaque white sample but no significant increase in viscosity was noticed. The sample became clearer upon further additions of plain P90G liposomes and was translucent at the end of the titration; no residual solid RSV was then detected.

The sample was let at least 5 min into the NMR magnet for temperature stabilization prior to recording the spectrum. The ^1^H NMR spectra were recorded using a 90° pulse, an acquisition time of 2 s, a relaxation delay of 10 s and 32 scans. Exponential apodization of the free induction decay with a line broadening factor (lb) of 2 Hz was applied before Fourier transform. The series of spectra recorded for increasing amounts of P90G liposomes are shown in Figure S9 *(region showing the signals of the liposomes; see article for the region of the RSV signals)*.

The molar concentration of the stock dispersion of liposomes, C_P90G-S_, was calculated according to equation SE1, considering a purity of 100 %, an average molecular weight (MW) of 775 g/mol *(*[*https://www.avantilipids.com*](https://www.avantilipids.com)*; catalog number 441601)* and assuming that the partial specific volume of the phospholipid bilayers ($\tilde{v}$) is 1 mL/g *(Huang et al.,* *J. Biol. Chem. 1971, 246, 2555-2560)*.

| $C_{P90G-S}=\frac{m_{S}}{MW}\times\frac{1}{\tilde{v} m_{S}+V_{water}}$ | (SE1) |
| --- | --- |

where *m_S_* is the mass of P90G in the stock dispersion of liposomes and *V_water_* is the volume of water (D_2_O) used to prepare the stock dispersion of liposomes. For a stock dispersion of liposomes comprising 100 mg of P90G for 1 mL of D_2_O, it yields C_P90G-S_ = 0.117 mol/L.

The calculated molar concentration and mass of P90G in the NMR sample are then given by equations SE2 and SE3.

| ${C_{P90G}=C}_{P90G-S} \times\frac{V_{Sa}}{V^{\circ}+V_{Sa}}$ | (SE2) |
| --- | --- |
| $m_{P90G}=C_{P90G}\times\left( V^{\circ}+V_{Sa} \right)\times MW$ | (SE3) |

where *V_Sa_* is the total volume of stock liposomal dispersion added in the NMR tube (ranging between 40 and 320 µL) and *V°* is the initial volume of sample in the NMR tube (500 µL).

The molar concentrations of P90G and RSV were determined by ^1^H NMR according to equation SE4, using respectively the integrals (*I*) of the TMA (*nH* = 9) and H-4 (*nH* = 1) signals and the integral measured for the signal of an external standard (*I_std_*) of known concentration (*C_std_*). The difference in NMR-tube inner volume was accounting for by a correction factor (*υ*). The corresponding mass was then obtained using the calculated total volume of the sample and the molecular weight, similarly to equation SE3.

| ${C=C}_{std} \times\frac{\frac{I}{nH}}{\frac{I_{std}}{{nH}_{std}}}\times\upsilon$ | (SE4) |
| --- | --- |

Our chosen standard is *1H*,*1H*,*8H*,*8H*-perfluoro-3,6-dioxaoctan-1,8-diol (purity > 98%, Fluorochem) because it can be used for both ^1^H and ^19^F NMR quantitative analyses (L. Fusaro, E. Locci, A. Lai, M. Luhmer, J. Phys. Chem. B 113 (2009) 7599–7605). It is soluble in D_2_O and gives a unique ^1^H NMR signal at about 4.0 ppm (triplet, ^3^J_H-F_ = 9.9 Hz, *nH_std_* = 4). The spectrum of the standard sample was recorded the same day, using exactly the same acquisition parameters as for the spectrum of the studied samples. The same processing was applied to both spectra. The integrals were determined by deconvolution using a home-made program developed in gnuplot. No overall baseline correction was applied to the spectrum but a local baseline correction was included in the deconvolution model. The line shape was assumed to be Lorentzian. The model used for RSV comprised a first-order triplet for H-4 and a first-order doublet for H-2,6 with a scalar coupling constant fixed to the value observed in the absence of P90G (^4^J=2.2 Hz), a local phase correction identical for both signals and a local quadratic baseline correction. The model used for the TMA signal of P90G comprised two singlet components, a local phase correction identical for both components and a local linear baseline correction. The deconvolution analysis is illustrated in Figure S10 for the spectrum recorded after addition of a total of 80 µL of liposome dispersion.

Deconvolution results for the TMA signal of P90G are shown in Figure S11. No significant chemical shift or linewidth variations were observed for the signals of RSV. The mean value of the full linewidth at half-height (broadening of 2 Hz due to exponential apodization included) is:

9.9 Hz, with a standard deviation of 0.1 Hz, for the signal H-4;

43.5 Hz, with a standard deviation of 0.3 Hz, for the signal H-2,6.

~

*Solvent*

***H****DO*

(e)

(d)

(c)

(b)

(a)

(f)

(g)

(h)

(i)

**Figure S9: Titration of an excess of solid RSV in D_2_O by plain P90G liposomes.**

Region of the ^1^H NMR spectra, showing the signals of P90G, recorded (a) in the absence of P90G liposomes and (b-i) after mixing with increasing amounts of P90G liposomes (300 K, 500 MHz). The sample used to acquire spectrum (a) comprised 500 µL of D_2_O and about 1 mg of RSV, which was mainly undissolved. Spectra (b-i) were recorded after repeated additions of 40 µL of a 100 mg/mL dispersion of plain P90G liposomes, up to a total addition of 320 µL. These spectra are superimposed without vertical offset in the lower part of the figure.


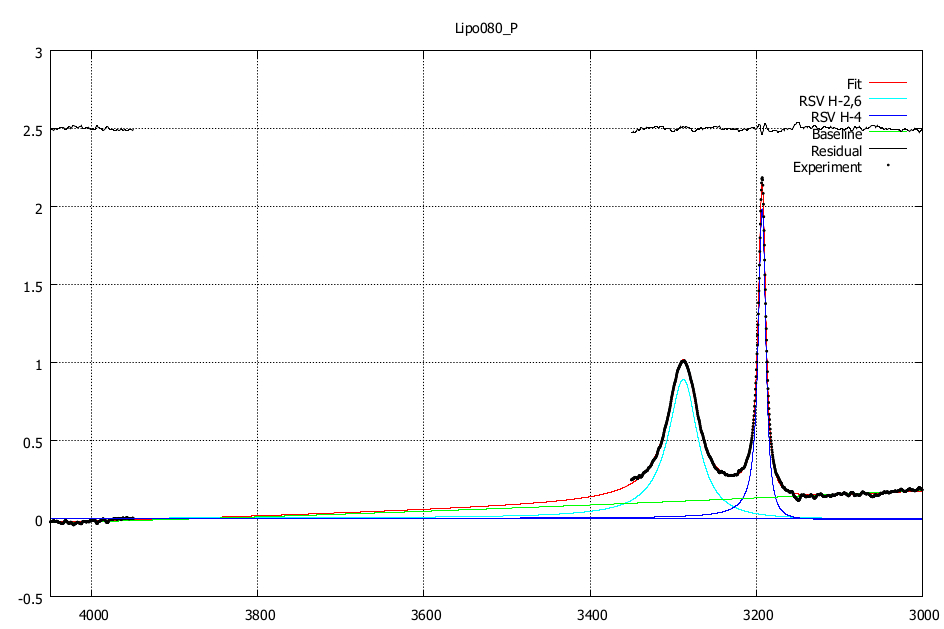


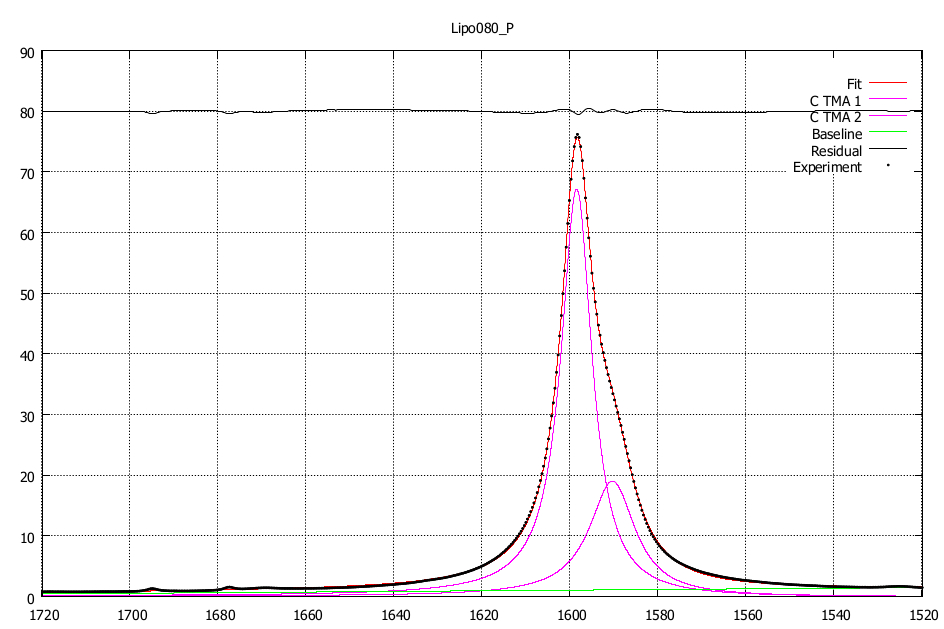


**Figure S10**: **Titration of an excess of solid RSV in D_2_O by plain P90G liposomes.**

Deconvolutions completed for the spectrum recorded after addition of a total of 80 µL of liposome dispersion; the horizontal scales are in Hz.

(above) Deconvolution of the RSV H-2,6 and H-4 signals.

(below) Deconvolution of the P90G TMA signal: TMA 1 corresponds to the most intense and narrower component and TMA 2 accounts for the shoulder observed at lower frequency.

|   (a) |   (b) |
| --- | --- |
|   (c)  (d)  TMA 1  TMA 2 |   TMA 1  TMA 2 |

**Figure S11**: **Results of the deconvolution of the TMA ^1^H NMR signal of P90G.**

(a) Comparison between the P90G molar concentration data measured by ^1^H NMR to the values calculated using equations SE1 and SE2. The confidence intervals correspond to twice the fitting errors.

(b) Integral ratio of the two components of the TMA signal (TMA 2 accounts for the shoulder observed at lower frequency, see Figure S10) ; mean = 0.42, standard deviation = 0.02.

(c) Frequency of the two components of the TMA signals.

(d) Full linewidth at half-height (Δν_½_) of the components of the TMA signal (broadening of 2 Hz due to exponential apodization included). TMA1: mean = 8.4, standard deviation = 0.2; TMA 2: mean = 12.8, standard deviation = 0.2.

The red data points correspond to a total addition of 240 µL and the blue data points correspond to two the subsequent additions of liposome dispersion.

**Samples of P90G liposomes prepared in the presence of various amounts of RSV.**

(e)

(d)

(c)

(b)

(a)

**Figure S12**: **Samples of P90G liposomes prepared in the presence of various amounts of RSV.**

^1^H NMR spectra of 100 mg/mL samples of P90G liposomes prepared in the presence of increasing amounts of RSV (300 K, 500 MHz): (a) in blue : 0 mg/mL (plain liposomes); (b-c) in black : 2 and 4 mg/mL; (d-e) in red : 6 and 8 mg/mL. The spectra were not corrected for baseline distortions. The horizontal green lines show the zero-intensity level of the spectra.

**Figure S13**: **Samples of P90G liposomes prepared in the presence of various amounts of RSV.**

^1^H NMR spectra of 100 mg/mL samples of P90G liposomes prepared in the presence of increasing amounts of RSV (300 K, 500 MHz): in blue : 0 mg/mL (plain liposomes); in black : 2 and 4 mg/mL; in red : 6 and 8 mg/mL. The spectra are superimposed without vertical offset.
